# Moyamoya phenomenon following clipping of intracranial aneurysm: case report

**DOI:** 10.1093/jscr/rjae841

**Published:** 2025-01-09

**Authors:** Masna B Inam, Ali Bakhsh, Mohammed Khattak, Arun Chandran, Jawad Yousaf

**Affiliations:** Department of Neurosurgery, The Walton Centre NHS Trust, Lower Lane, Liverpool L97LJ, United Kingdom; Institute of Systems, Molecular and Integrative Biology, University of Liverpool, Brownlow Hill, Liverpool L697ZX, United Kingdom; Department of Neurosurgery, The Walton Centre NHS Trust, Lower Lane, Liverpool L97LJ, United Kingdom; Department of Neuroradiology, The Walton Centre NHS Trust, Lower Lane, Liverpool L97LJ, United Kingdom; Department of Neurosurgery, The Walton Centre NHS Trust, Lower Lane, Liverpool L97LJ, United Kingdom

**Keywords:** aneurysm, clipping, moyamoya, subarachnoid, headache

## Abstract

Subarachnoid haemorrhage from aneurysmal rupture is a common emergency in neurosurgery. Depending on aneurysm position, morphology, size, associated clot, and symptoms, it is either managed by endovascular occlusion or by clipping. Here we report the first known case of secondary Moyamoya phenomenon following the clipping of a supraclinoid internal carotid artery Aneurysm. After making a complete recovery following clipping, this patient developed headaches 6 years later. Angiographic imaging revealing Moyamoya phenomenon characterized by total distal arterial occlusion and development of anastomotic collaterals. This phenomenon may be caused by neuroinflammation and suggests a tailored neuroimaging follow-up is required for such patients.

## Introduction

Moyamoya, meaning “hazy puff of smoke” in Japanese, is a rare, chronic, disease characterized by a progressive stenosis of the terminal portion of the internal carotid arteries and subsequent development of a network of abnormal collateral vessels. It is usually idiopathic but can develop secondary to an underlying disorder such as atherosclerosis, vasculitis, Down’s syndrome, sickle cell disease, William syndrome, or neurofibromatosis, or may occur after brain radiation therapy [1, 2]. Moyamoya causes hypoperfusion symptoms that clinically manifest as headaches, stroke phenomena, and territory dependent neurological deficits [3]. We describe the first ever report of Moyamoya developing after the surgical treatment of a cerebral artery aneurysm.

## Case report

**Figure 1:**
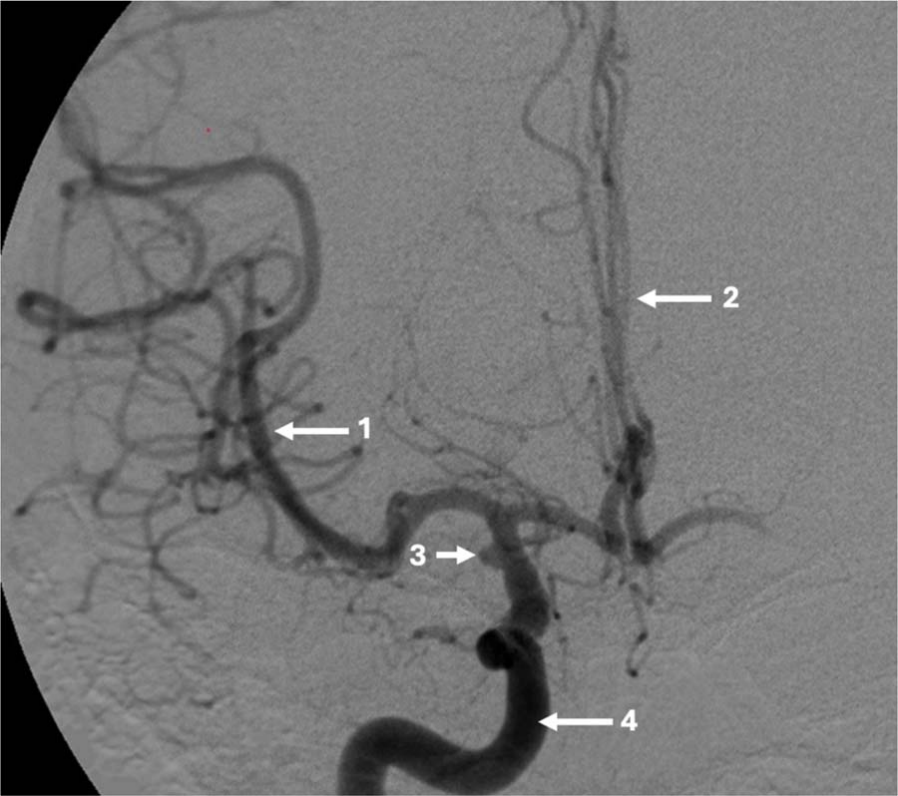
Pre-operative CT angiogram demonstrates normal appearances of the (1) MCA, (2) ACA with, (3) laterally projecting terminal carotid sidewall aneurysm. (4) R ICA.

**Figure 2:**
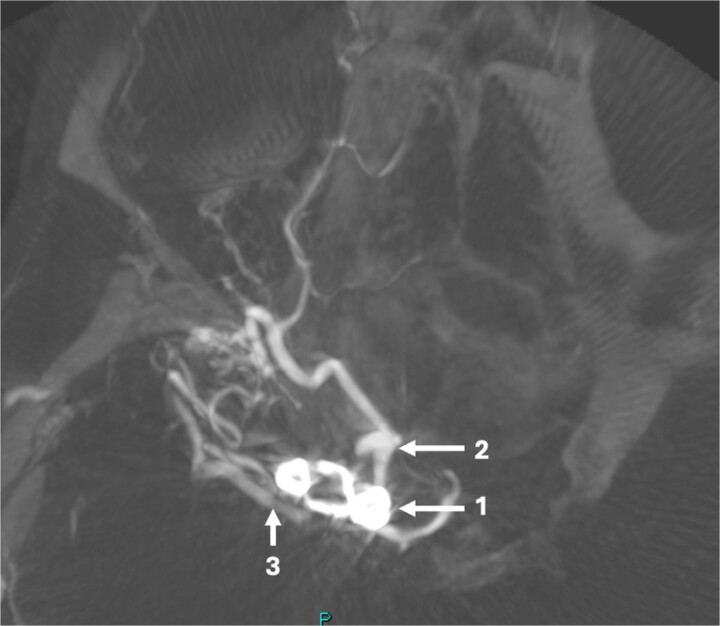
Axial post-operative CT angiogram demonstrating the (1) encircling clip occlusion of the terminal internal carotid artery aneurysm with a relatively normal calibre of (2) A1 and, (3) M1.

A 43-year-old lady, with known hypertension, presented with severe and sudden onset headache in 2010. Initial computed tomography (CT) head confirmed a diffuse subarachnoid haemorrhage (images not available) and a subsequent CT angiogram showed a right supraclinoid internal carotid artery (ICA) aneurysm (Fig. 1). CT Angiogram also showed an unruptured left posterior communicating which was thought not to be related to this patient’s presentation and managed conservatively with serial imaging. The patient underwent emergency craniotomy on the same day where an encircling Sundt clip was applied to secure the aneurysm. She made a full neurological recovery and was discharged from hospital. Post-operative day 1 CT angiogram demonstrated relatively normal calibre of the right A1 portion of anterior cerebral artery (ACA) and M1 portion of middle cerebral artery (MCA), in the terminal carotid region (Fig. 2).

At 2 years, a follow-up CT angiogram was reassuring. After a symptom free period of 6 years after the initial operation, this patient started experiencing intermittent holocranial headaches, severe enough to necessitate presentation to Emergency Department. CT scans did not reveal any new abnormalities. In 2018, after multi-disciplinary discussion for the ongoing headaches, a digital subtraction angiogram (DSA) was offered to the patient to investigate the status of the clipped aneurysm and the untreated, unruptured posterior communicating (PCOM) aneurysm as a cause of her headaches.

**Figure 3:**
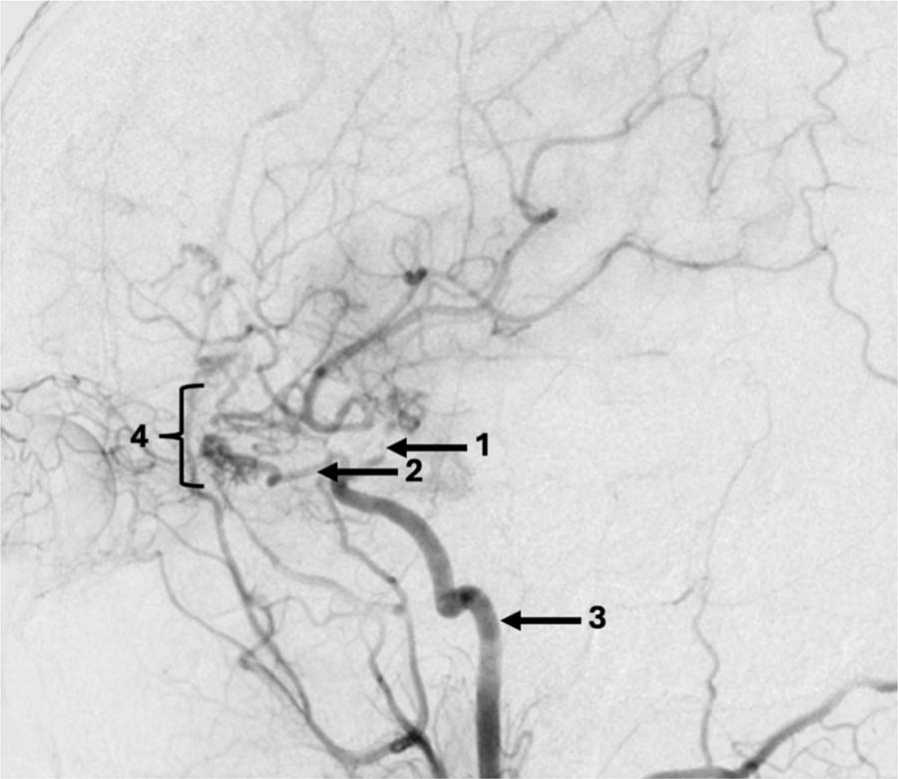
Sagittal DSA demonstrating (1) Clip is in position across the terminal carotid. The frontal branch of the right MCA is supplied by collaterals from the (2) right ophthalmic artery (hypertrophied recurrent meningeal artery), right middle meningeal artery and right superficial temporal artery (*via* craniotomy site as pial synangiogenesis). There is collateral vessel formation consistent with Moyamoya phenomenon (4). (3) ICA. The A1 is not opacified likely due to the anterior communicating (ACOM) collateralisation.

The DSA showed significant narrowing of the ACA and complete occlusion of the MCA distal to the clipped and fully occluded aneurysm. The right recurrent artery of Heubner, arising from A1, was hypertrophied and supplying the medial lenticulostriate territory. There was diffuse small vessel formation in this same area (Fig. 3). This DSA suggested the progressive distal stenosis secondary to the surgical clip led to anastomotic collateral formation, consistent with a radiological and clinical Moyamoya phenomenon. Patient’s symptoms were managed conservatively. Subsequent CT angiogram in 2018, 2021, and 2023 showed no further progression of the Moyamoya phenomenon.

## Discussion

This is the first known report of Moyamoya phenomenon developing after surgical clipping of an aneurysm. The clinical presentation and imaging findings show a lag of 6 years after primary clipping. The aetiology of distal narrowing and compensatory collateral vessel formation is debatable. Given the involvement of pre-existing inflammation in aneurysmal wall growth and rupture, the subsequent introduction of a foreign material (the surgical clip) may have continued the inflammatory response. Progressive neuroinflammation may lead to the vasocculsion seen in neuroimaging. Inflammation mediated angiogenesis, through vascular endothelial growth factor and fibroblast growth factor-1, may contributed to collateral vessel formation and the Moyamoya appearance [4]. The alternative hypothesis is a delayed hypersensitivity reaction. Sundt clips are made from a cobalt-based alloy that contains nickel which is a common cause of sensitivity. However, a hypersensitivity reaction is thought to be less likely given the lack of cerebral oedema and the long delay before onset of symptoms.

Neuroinflammation may be the common factor in secondary Moyamoya phenomenon. Moyamoya secondary to occlusion of supraclinoid ICA following an ischemic stroke in the MCA territory is now well described [5]. Stroke pathology prominently involves hyperactivation of major biological pathways, such as PI3K/AKT/mTOR, which disrupt cell adhesions, promote fibroblast migration and cell proliferation [6], all which may contribute to intimal vessel wall morphological changes and eventual vasoocclusion. In this present case, the inflammatory response was prolonged with the addition of an encircling metal clip.

This patient suffered headaches most likely due to decreased regional blood flow. The headaches were intermittent and in the absence of a deficit and non-progressing imaging findings, it was agreed to continue with active observation. The key lesson from this case is to consider a formal angiography study, if CT angiogram cannot explain neurological symptoms, to rule out Moyamoya phenomenon. These patients may need a longer follow-up to monitor the development of any neurological deficit, necessitating further intervention. Indeed, this case gives further evidence to continue routine vascular imaging follow-up in all patients for at least 5 years.

## Declarations

There are no financial conflicts of interest to declare for the authors. This report adheres to all local trust protocol ethical standards. No funding was sought for this study.
